# The relationship between geographical and social space and approaches to care among rural and urban caregivers caring for a family member with Dementia: a qualitative study

**DOI:** 10.1080/17482631.2016.1275107

**Published:** 2017-01-17

**Authors:** Kethy Ehrlich, Azita Emami, Kristiina Heikkilä

**Affiliations:** a Department of Neurobiology, Care Sciences and Society, Division of Nursing, Karolinska Institute, Huddinge, Sweden; b Biobehavioral Nursing & Health Systems, School of Nursing, University of Washington, Seattle, WA, USA; c Aging Research Center (ARC), Karolinska Institute, Stockholm, Sweden; d Department of Health and Care Sciences, Faculty of Health and Life Sciences, Linneaus University, Kalmar, Sweden

**Keywords:** Dementia, family caregivers, qualitative research, rural, urban

## Abstract

Knowledge about family caregivers in rural areas remains sparse. No studies to date have addressed the sociocultural aspects in caregiving, thus neglecting potentially significant data. This study aimed to explore and better understand family caregivers’ experiences in rural and urban areas and the sociocultural spheres that these two areas represent. How do family caregivers approach their caregiving situation? A hermeneutical approach was chosen to uncover the underlying meanings of experiences. Open-ended in-depth interviews were conducted. The ontological and epistemological roots are based on hermeneutic philosophy, where a human being’s existence is viewed as socially constructed. The study followed a purposeful sampling. Semi-structured in-depth interviews were conducted with 12 rural and 11 urban family caregivers to persons with dementia. These were then analyzed in accordance with the hermeneutical process. The findings provide insight into the variations of family caregiver approaches to caregiving in rural and urban areas of Sweden. There seemed to be a prevalence of a more accepting and maintaining approach in the rural areas as compared to the urban areas, where caregiving was more often viewed as an obligation and something that limited one’s space. Differences in the construction of family identity seemed to influence the participants approach to family caregiving. Therefore, community-based caregiving for the elderly needs to become aware of *how* living within a family differs and how this affects their views on being a caregiver. Thus, support systems must be individually adjusted to each family’s lifestyles so that this is more in tune with their everyday lives.

## Introduction

### Family caregiver experiences

A substantial body of research has documented the various experiences of family caregivers to the frail elderly, especially those with dementia. Research has generally shown high levels of caregiver burden and negative health effects across geographical regions, cultural groups and healthcare delivery systems (Haro et al., 2013; Pinquart & Sörensen, 2003; Torti, Gwyther, Reed, Friedman, & Schulman, 2004). Problems related to caregiving are experienced through all stages of the illness, with a preponderance of physical demands and issues in the later stages (Haro et al., 2013; Zwaanswijk, Peeters, Van Beek, Meerveld, & Francke, 2013). However, family caregivers revealed coexisting positive benefits and negative effects of being the caregiver for a family member (Cohen, Colantonio, & Vernich, 2002; Ehrlich, Boström, Mazaheri, Heikkilä, & Emami, 2015; Habermann, Hines, & Davies, 2013; Shim, Barroso, & Davis, 2012); these included feelings of grief together with a feeling of personal growth throughout the caregiving process (Ott, Sanders, & Kelber, 2007).

### Community health resources in urban and rural Sweden

In Sweden, the majority of elderly care is carried out by relatives and their contribution has increased since the 1980s. Both urban and rural communities, to varying degrees, offer support to relatives who take care of a close relative with dementia disease. For example, they can be given support in the form of relief care where the dementia patient is then cared for by a dementia nurse for a number of hours. Other forms of support include counseling and practical support, such as short term living solutions for persons with dementia or being invited to participate in meetings with other caregivers in the same situation. They are therefore provided with the opportunity to share experiences and obtain support, encouragement, education and help in dealing with everyday situations (The National Board of Health and Welfare, 2012). However, there are several municipalities that still do not meet the requirements of providing information to relatives regarding the support measures that are available and there are also shortcomings in identifying and investigating the support needs of families (The National Board of Health and Welfare, 2014).

### The caregivers’ place of residence

The majority of studies focusing on the family caregivers’ situation are based on the assumption that the place of residence does not play a significant role on caregiver experiences and most studies have previously been conducted in larger cities (Alvira et al., 2015; Evans & Lee, 2014; Shim et al., 2012). When it comes to studies in rural areas these usually focus on caregiver experiences, the need for and use of services, and problems with service delivery (Bedard, Koivuranta, & Stuckey, 2004; Dal Bello-Haasm, Cammer, Morgan, Stewart, & Kosteniuk, 2014; Innes, Morgan, & Kosteniuk, 2011; Lucke et al., 2008; Pinquart & Sörensen, 2005) and these studies seldom focus on how to contemplate the caregivers’ experiences. However, Årestedt, Benzein, Persson, and Rämgård (2016) showed how social relations and activities in families over time were intertwined and connected with geographical place, which created feelings of respite in families living with chronic illness.

In this study, we assume that people construct their everyday life with others living in the same social context. Behaviors and attitudes then become internalized, like unwritten rules which are constantly re-created (Berger & Luckmann, 1966/2007) and serve to construct the culture people live in (Ehn & Löfgren, 1982). The underlying assumption is that people in rural and urban regions construct their lives differently, which may influence family caregiving when a family member requires care. We are concerned with a sociocultural “place” that is defined by geography and as a dynamic social space (Staeheli, 2003). This study focuses on these two aspects.

Many recent studies on the cultural aspects of caregiving are related to differences among ethnic minorities (Kong, 2007; Losada et al., 2006; Sun, Ong, & Burnette, 2012). These studies usually found strong family ties among ethnic minorities, and the ideal model of taking care of the elderly was believed to be a family responsibility. Conversely, Anngela-Cole and Hilton (2009) found more positive attitudes towards caregiving among groups from Western oriented, individualistic cultures than from groups with traditional family-focused belief systems based on the possibility to choose to care. However, an ethnic perspective risks overlooking what may be significant variations within ethnic groups in regard to caregiving attitudes. In a Swedish context, Winqvist (1999) showed that despite many similarities in family caregiver experiences, the approach to caregiving varies in different sociocultural spheres. Family caregivers from the working class were more family-focused when caring for a parent needing assistance, compared to family caregivers from other, more individualistic social classes. Thus, the culture-bound feature of the caregiving experience seems to be a complex issue, with several aspects governing the experience.

As most studies on family caregiving are conducted in larger cities, our knowledge about family caregivers in rural areas remains sparse (McKenzie, McLaughlin, Dobson, & Byles, 2010). Existing studies show less use of formal services and a greater family involvement in rural areas (Pinquart & Sörensen, 2005; Wenger, Scott, & Seddon, 2002). Bedard et al. (2004) found that there was less access to formal support in the rural context, while caregiver burden, health status and health behavior were found to be similar in urban and rural areas. On the other hand, Lucke et al. (2008) showed that even when appropriate services were available, the caregivers from both areas did not fully utilize these services. A review by Turner Goins, Spencer, and Byrd (2009) found that there was no clear and consistent rural/urban difference in caregiving, a conclusion supported by McKenzie et al. (2010). However, in Sweden, Nordberg (2007) reported that elderly people in urban areas used three times more community-based support than elderly people living in rural areas, although those being taken care of in rural areas showed more cognitive decline than those in urban areas. A plausible explanation could be the fact that there is a greater family involvement in rural areas. In order to develop targeted support to family caregivers, greater knowledge is needed regarding the individual social and cultural family lifestyles in rural as opposed to urban areas regarding caregiving. The aim of this study is to explore and better understand the interrelationship between the caregiving experiences of family caregivers and the sociocultural sphere that urban and rural areas represent. How do family caregivers approach their caregiving situation in rural and urban areas?

## Method

A hermeneutical approach was chosen to uncover the underlying meanings of experiences and open-ended deep interviews were conducted for the data collection (Gadamer, 2002; Ödman, 2007; Ricoeur, 1993). The ontological and epistemological roots of this study are based on hermeneutic philosophy, where each person’s existence is considered to be socially constructed, in a dynamic process involving both socialization in the family culture and internalization of cultural norms and the value system of the surrounding society (Berger & Luckmann, 1966/2007).

This social construction is based on a naturalistic approach, where the articulation of the taken-for-granted aspects is embedded in the linguistic and symbolic expressions of daily life. Therefore, the narratives expressed by the family caregivers in this study are based on their everyday lives and other aspects of their lives that are taken for granted (Ödman, 2007).

In order to uncover the family caregivers’ approaches to caring for family members with dementia, interviews were conducted which focused on the caregivers’ narratives about their life situation. The analysis process was conducted with a hermeneutic approach using a circular process, guided by Ödman (2007), whose methodological foundation is based on Ricoeur’s (1993) and Gadamer’s (2002) philosophy.

### Sample and participants

#### Study sites

The study was conducted in Sweden, in five of the 26 municipalities of Stockholm County (urban areas), with a population density of 451.1–4796.2 inhabitants per km^2^, and in three of 15 municipal areas of the province of Dalarna (rural areas) with a population density of 1.1 to 13.1 inhabitants per km^2^ (Statistics Sweden, 2014). The study sites were chosen through contacts that helped during the recruitment process.

#### Recruitment of participants

The inclusion criteria for participants were as follows: cognitively intact persons who had the primary care responsibility for a family member diagnosed with dementia disease for two or more years and who were living at home. The participants were recruited through contacts with the managers of community-based daycare settings and dementia specialist nurses. A purposeful sampling procedure was used to reach persons that were willing to speak openly about their situation. A letter with information about the study was sent to the managers and nurses in eight municipal areas of Dalarna, and to 11 municipal city districts of Stockholm. Shortly after, the first author contacted the managers by telephone with a request to deliver a letter of information about the study to candidates who met the inclusion criteria. Those caregivers who volunteered to participate called the first author directly, or gave their permission for her to call them.

A total of 31 family caregivers were contacted by the first author. The participants were informed about the aim of the study, and the question areas. They were also informed that participation was voluntary, that they could cease participation at any time, and that their identity would not be revealed. Four family caregivers in each of the two geographical areas declined participation. The final study cohort contained 23 family caregivers; 12 from Dalarna and 11 from Stockholm; 18 were partners and five were adult children (Table 1).

During the interviews, we found that five persons being cared for by participants in the rural areas lacked a formal diagnosis of dementia. However, all were assessed by a specialist nurse in dementia and received municipal support as if they had been diagnosed. The participants remained in the study because their experiences did not differ from the other family caregivers of diagnosed family members.

### Data collection

All interviews were conducted by the first author in locations chosen by the participants, mainly in their own homes in 2006–2009. Open-ended interviews were conducted with the use of an interview guide, which included a short life history, social network, family life, daily life and work, and the experience of living with and caring for a relative with dementia. The participants were encouraged to talk freely about each topic. To acquire a clearer and more robust understanding of their thoughts, follow-up questions were asked. All interviews were tape recorded after verbal agreement was provided by the participants. The interviews lasted for 50–90 min and all were transcribed verbatim. Interviews were conducted individually with the primary caregiver while the family member with dementia was cared for by other persons or when they were resting. In this study, member checking was not carried out with transcribed interviews since word-for-word accounts, pauses and repetitions can be experienced as negative by participants (Kvale, 1997).

### Ethical considerations

Informed consent was obtained from the study informants and the study was approved by The Central Ethical Review Board at Karolinska Institute (Dnr 03–069).

### Data analysis

The interviews were analyzed using Ödman’s (2007) hermeneutical approach. Throughout the entire analysis process, the authors’ pre-understanding of personal experiences and knowledge was discussed. This was acknowledged as both an obstacle and a resource during the process. The aim was to be as open as possible regarding the underlying meaning within each text and to facilitate the authors in understanding the content of the text in a new way (Ödman, 2007). Consistent with Ödman (2007), the analysis was carried out by two of the authors (KE and KH), thus enhancing reliability of the analysis by enabling a discussion of different aspects, contradictory information and interpretations. Throughout the process, the meaning of the material was constantly examined and challenged. It was then discussed within the research group for critical examination and the integration of contradictory or complementary interpretations (Ödman, 2007).

All interviews were read several times. Notes were made about how the family members approached their situations. Each interview was then read more thoroughly in order to grasp the individual “sound” of each one. It began with the interviews conducted in the rural areas, followed by those from urban areas. To illuminate the unvoiced meaning – the “surplus of meaning” – Ricoeur (1993) suggests a distancing, questioning, and critical approach. Therefore, the next phase was to pose questions to the interview text about the family members’ approaches in relation to their own situation, such as how family caregivers speak about it in relation to themselves, toward their spouses or parents, their family, their work or previous work and to the society around them. A summary, including a preliminary interpretation of the meaning of each interview text, was created and reconsidered. This phase resulted in a better understanding of the caregivers’ situations.

The material was then read from each individual’s perspective to uncover the meanings and to assign meanings in a constant dialectic act by reading the summaries and returning to the original text to confirm or reject our interpretations (Ödman, 2007). This phase resulted in three tentative categories that best described how family caregivers approached their life situations. Each category uncovered certain characteristic patterns in the participants’ approaches. The differences and similarities between the urban and rural areas were analyzed in detail with the use of similar processes, which resulted in uncovering a tentative difference between rural and urban caregivers’ approaches to their life situations related to living with a family member with dementia. These tentative differences were then checked and validated against the entire corpus of material, to acquire an understanding of whether the manifested differences were related to a pattern based on the participant’s location of residence or simply reflected individual or idiosyncratic differences. Finally, a matrix was created. It included each individual urban and rural family caregiver labeled with the most significant of the three approaches together with the individual and collective dimensions. Variations in discrepancies and commonalities in these respective categories were further scrutinized, until a coherent picture emerged.

As researchers we posed questions to the text, such as how, and in which way, did the participants talk about their situation in regard to themselves or in regard to their ill partner or spouse or to the family as a whole. Other aspects such as the caregivers work or earlier work and their surrounding community were also posed. The material was then read from each participant’s own perspective in order to uncover the meanings and assign meanings in a constantly ongoing dialectical act with the original text (Ödman, 2007). This resulted in three categories that best described how family caregivers approached their life situations. These were labeled as: *accepting the course of life*; *preserving normalcy*; and *fulfilling obligations*. Each category uncovered certain characteristic patterns in the participants’ approaches. Finally, an analysis was made, by looking at the similarities and differences between urban and rural narratives (Table 2).

## Findings

Most of the 23 family caregivers in both areas were older female spouses (Table 1). Family caregivers from both areas stressed a sense of belonging to their surrounding communities, and thus this seemed to be a representative sample from each environment.

**Table 1.** Sample demographics.

Characteristics	*n* Rural (12)	*n* Urban (11)
Family caregiver age group		
30–65 years old	4	4
66–75 years old	4	6
> 75 years old	4	1
Median age (range)	71.5 (48–83)	69 (33–80)
Family caregiver gender		
Male	3	2
Female	9	9
Recipient age group		
30–65 years old	1	3
66–75 years old	1	2
> 75 years old	9	6
Median age (range)	80 (61–87)	77 (63–85)
Median age non- diagnosed (range)	84 (81–87)	
Relationship of caregiver to recipient		
Spouse	9	9
Adult child	3	2
Family caregiver maximum education level		
Elementary school	5	2
Vocational school	3	6
High school		
University/college	4	3
Recipient gender		
Male	7	8
Female	5	3
Average length since diagnosis		
1–5 years	4	9
6–10 years	3	2
Non- diagnosed	5	
Recipient maximum education level		
Elementary school	5	3
Vocational school	4	2
High school		4
University/college	2	1
Missing	1	1

**Table 2.** Characteristics of each theme.

Theme	Rural	Urban
**Accepting the course of life**	The illness as a natural part of life when you get older, even if it feels hard. Make daily life work.	The illness as something you have to cope with and is related to a positive view of life. Take one day at a time.
	*“Surely, it would have been easier if it had been… ok. But you cannot have everything.”*	*“And I am anyhow a relatively positive person and I try and we try to do the best we can.”*
**Preserving normalcy**	Focus on the family or the couple. Togetherness. The caregiver is a part of the whole. Caregiving as part of the whole; Family, surroundings, friends and the landscape.	Focus on the individual. Side-by-side. The caregiver is an individual as a part of the whole. Caregiving as a decision made by the individual, which also includes reflection and focus on oneself as a caregiver.
	*“We have helped each other with everything. We have always worked as a team. But as I said, we have always kept the family together.”*	*“When I am with John, it is so relaxing that I cannot experience this anywhere else. Some people pity me but it is almost a gift that when I am stressed and I sit here and John sits there and we listen to music…”*
**Fulfilling obligations**	A conflict between different family demands or shifting roles and power in the relationship. Limited personal independence is not primarily in focus	Dreams and plans for the future are crushed to the ground. Limited personal independence is stressed.
	“*You constantly live with a guilty conscience, that you should have helped more and whatever you do you think that you should have been in another place. When I am at my parents I think that I should be at home helping my family and when I’m at home I feel I should be there instead.”*	“*At the same time, I have been a person who is used to freedom of movement and now I feel very much tied down /–/ I am very grateful to these people who come* (the home care assistants). *At the same time it feels like a hotel business. I am not alone in my own home with my own husband.”*

The experiences of living close to a relative with dementia showed great commonalities between family caregivers in both urban and rural areas. These included a psychological burden of grief, worry, deep frustration and feelings of loneliness combined with positive aspects, such as an emotional closeness. All participants stressed the importance of family and friends irrespective of the geographical setting.

Based on their stories about the family’s or the couple’s life history and the actual situation, three ways of approaching caregiving were crystallized which were common for both areas: *accepting the course of life*, *preserving normalcy* and *fulfilling obligations*. However, rural family caregivers seemed to more frequently take the approach of accepting life as it came and they tried to maintain a sense of normalcy as well as they could. Urban caregivers however expressed more frustration and felt that they had lost their personal independence.

Differences were found in the narratives of rural and urban caregivers when they talked about themselves and the family/the couple in relation to how their earlier lives had been as compared to the lives they now lived. In rural areas the caregivers tended to talk about themselves as a part of a family or couple, often referring to themselves as “we” rather than “I”. There was a clearer sense of “togetherness”. In urban areas, the caregiver’s sense of individuality was at the forefront and this was seen distinctly with the use of the word “I” as an independent person who needed time for oneself. Individuality seemed to become more visible when caregiving was seen as an obligation that had to be fulfilled irrespective of the geographical area.

### Accepting the course of life

The most common approach taken by rural caregivers was to accept the course of life. It implied an acceptance of life as it was or had become, including the manifestations of the illness and the challenges that arose from this.

*In rural areas* accepting the course of life meant a slow adjustment to life at home. Aging and illness were accepted as natural parts of life, or as one daughter (51 years old) put it: “Well, if you are 82, so well, it is no big deal so to say” (that the parent had got dementia). An 80-year-old husband said: “Surely, it would have been easier if it had been … ok. But you cannot have everything.”

The rural family caregivers seemed to approach the situation as an inevitable part of their lives and they accepted that this was how things would be now. Feelings of irritation and anger were muted, although a seldom-voiced, quiet sense of grief was still present.

Most of the family caregivers felt that closeness to one’s family and to the community provided satisfaction and made it easier to accept what life had become. Despite their feelings of insecurity regarding the future they were prepared for the changes to come. Some of the family caregivers felt that their current daily lives were working rather well, as illustrated below by one couple who continued with their everyday activities to the best of their ability.

Well we do it together now as well /–/when we change curtains and things like that we help each other and she does one thing and I do another, so she won’t have to climb up anywhere /–/and… well, she is out in the garden and well, we help one other there too so eh … I usually joke and say that she plants stuff and I cut it down. (Husband, 71 years old)

In urban areas, similar aspects were expressed by the family caregivers but these were based more on their positive attitude to life. The spouses had lived together in a close relationship and had experienced their share of ups and downs, but they always had a private sphere of their own. Trepidation about the future was mixed with a hopeful attitude about “the possibility” of finding bright spots in life, which seemed to represent the caregiver’s individual strength.

And I am anyhow a relatively positive person and I try and we try to do the best we can. /–/When he has a really bad day, then there is not so much … one can do. Then I try not to think and ponder on it that much. Because then I also get so … depressed. (Wife, 69 years old)

### Preserving normalcy

Common to both rural and urban family caregivers was the fact that they wished to preserve their spouse or parent as a person, both as they once had been and as they were now. They also wanted to make it possible for the person with dementia to live at home for as long as possible, as a family member.

In rural areas, the family caregivers tried to maintain a sense of normalcy by attempting to preserve the couple’s and family’s common history, and by sharing memories of the past together in order to emphasize a feeling of unity within the family. This preservation was expressed through feelings of love and togetherness, as matrimonial commitments, or as filial piety with respect for one’s parents. Preserving a normal life was about preserving a sense of kinship with one’s spouse, family and friends and with the local community. The nuclear family and the couple as a specific unit had a special value for two of the family caregivers. In their narratives, all parts of the family member’s lives were intertwined. They formed a web where all threads had, in some way, a meaning to the family, in order to preserve their life together. The past and the present flowed together and gave meaning to their present lives.

Everything was conducted for the benefit of the family and they worked side by side, conducting their own work tasks as a part of a shared goal and building unity within the family:

We have helped each other with everything. We have always worked as a team. But as I said, we have always kept the family together. Very, very much … and we are so used to it. (Wife, 74 years old)

Through friends’ visits and by asking the home care assistants to attend to the morning coffee, a husband was made a part of this unity and was maintained as a person of worth.

Several of the caregivers tried as much as possible to let their spouses share household tasks, to the best of their abilities:

I cannot do this when it comes to kneading the dough. That’s for my missis. We help each other … I pick up the dough, put it on the … well we have a baking board that we, I pull out. Now Mary wants it on the table where she sits, then I’m at her side when she cuts up the pieces, slices the buns. And then, when we do flat long-shaped buns … It is Mary that braids the bread. (Husband, 83 years old)

In urban areas, preserving normalcy was mainly based on maintaining a sense of community with one’s spouse or parent as an individual, and being aware of the family caregiver’s own feelings. The narratives gave descriptions of each person’s needs, but at the same time they expressed a desire to preserve the family and the couple. This was expressed in different ways, such as “I” help “you” or “I” please “you” or “you” give “me,” for example, when a husband helped his ill wife to visit church, even though the husband was not religious or interested himself.

Some of the family caregivers tried to preserve their day-to-day life together with the impaired person by using wordless communication during small moments of lucidity.

When I am with John, it is so relaxing that I cannot experience this anywhere else. Some people pity me but it is almost a gift that when I am stressed and I sit here and John sits there and we listen to the music, and he teaches me so honestly and looks at me. And this calmness, it is so relaxing. (Wife, 59 years old)

For one of the daughters, caring for someone was an active choice, both as a means of preserving unity within the family and a way to protect the parent as the person he was now and the memories of what he had done for her. However, she felt frustrated that there was no social acceptance among her friends regarding taking care of one’s parent:

Certainly, people can say like, “Yes, it’s great, it’s great that you take care of your dad, that’s great.” But actually they think that it is … yes, but “do something else instead” … (Daughter, 33 years old)

### Fulfilling obligations

In both areas, some of the family caregivers set about caring with tension. However, this was more explicit and expressed more frequently in the urban areas. It was a task that had to be done, and they felt that they could not do what they otherwise had in mind or had planned. This sense of obligation resulted in feelings of being limited and it affected their sense of independence. Adult daughters in both areas felt trapped between conflicting expectations and the demands of taking care of a frail family member.

*In the rural areas*, two adult daughters described trying to meet the needs of their own families while fulfilling the additional obligation of attempting to keep their parents’ lives together.

You constantly live with a guilty conscience, that you should have helped more and whatever you do you think that you should have been in another place. When I am at my parents I think that I should be at home helping my family and when I’m at home I feel I should be there instead. (Daughter, 48 years old)

They struggled with unspoken family ideals expressed both as an inner picture of their own aspirations and also as an invisible pressure from society in general, that expects them to take care of a family member. Their own wishes in life were subordinated to caring for others:

Of course sometimes when it feels stressful, now I have one hour to manage and I have to manage to… But also, I know that I have been very careful with the weekly cleaning as I know that they get desperate if it’s not well cleaned before weekend and that’s why I try. (Daughter, 48 years old)

One rural daughter explicitly expressed an aversion to taking care of a parent who had never shown her closeness. Despite this she took on the role of caregiver:

And then I have to be the good daughter who is expected to provide support and be reliable and happy and capable all the time, Yes. /–/I feel an obligation from somewhere that I have to deal with everything. /–/or/–/they are like that here in the countryside, it is the daughters who are expected to be there and take care of them. (Daughter, 65 years old)

In the urban areas, most of the family caregivers experienced frustration and feelings of having to sacrifice future plans. Several family caregivers had always had interests of their own, separate from those of their spouse or family. They were now frustrated about the restriction of those interests due to the additional responsibilities of caregiving.

She has always taken care of her things and I have taken care of mine and … well then we have shared the rest. And if she has wanted to go out with her friends /–/and … I have been out with my friends. And that has never been any trouble. But now … You have to clean, to make the bed, to wash and … wash her clothes, you just have to … oh, and it just goes on and on … so when she goes to bed you just sit there alone. (Husband, 74 years old)

Several other family caregivers expressed feelings of isolation and imprisonment, since their present lives were so different from their earlier lives where they had lived independently yet together. For them, their present life limited their personal space and independence, and tied them to their homes, both physically and mentally.

At the same time, I have been a person who is used to freedom of movement and now I feel much tied down /–/I am very grateful to these people who come (the home care assistants). At the same time it feels like a hotel business. I am not alone in my own home with my own husband. (Wife, 73 years old)

## Discussion

Many of the aspects of being a family caregiver identified in this study were similar to those found in earlier studies, such as stress and frustration, a loss of future dreams, meaning and joy (Butcher, Holkup, & Buchwalter, 2001), grief (Ott et al., 2007), empathy and compassion (Shim et al., 2012), loss of independence (Paun, 2003), and companionship and reciprocity (Evans & Lee, 2014), learning to handle the forgetfulness and adjusting to limited social relations (Todres & Galvin, 2006).

However, the findings of the present study provide insight into the variations in family caregiver attitudes when comparing rural and urban areas in Sweden. Attitudes such as an *acceptance of the course of life*, *preservation of normalcy* or as a *fulfillment of obligations*. These attitudes were expressed both as feelings regarding the help and support of the person with dementia, and also included the practical organization of daily life. All three of the above-mentioned approaches were represented in both areas, but there was a clear preponderance of a more accepting and preserving attitude in the rural areas as opposed to rural areas where caregiving was seen more as fulfilling an obligation. Kirby et al. (2016) found that rural residents had a more resilient attitude to death and fewer life supporting interventions were carried out, according to this review of palliative end of life care. This can be interpreted as an acceptance of the course of life. However, unlike the previously mentioned study, our research did not find the same positive role of social networks among rural residents.

Throughout the three approaches, another pattern seemed to permeate the narratives; the use of “we” and “I”, both linguistically and in the narratives of daily life and family history, which could imply different social constructions of what a family is and how it functions, or should function. The major elements of collectivistic cultural ideals seemed to be more dominant in the rural areas, while individualistic cultural ideals seemed to dominate the urban areas. In this study, as in Pyke and Bengtson (1996), family caregivers seemed to have a predominant orientation to either collectivistic or individualistic cultural ideals, but in practice, their orientation was a varying mixture and dependent on the situation. It may be possible to view old rural traditions as subordinating the self and personality and creating communion through labor (Frykman & Löfgren, 1979) and persisting in ways of expressing “we,” while in contrast, urban traditions emphasize individual freedom (Ehn & Löfgren, 1982). In our study, the same municipal support measures seemed to be interpreted differently depending on the focus. For the rural dwellers, the construct of the family as a unity was able to readily embrace and find a comfortable place for home care assistance; in the urban construct, the same assistance became a trespass and disturbance of personal integrity and freedom. It seems that support measures provided to caregivers were interpreted differently in rural as opposed to urban cultures. In rural areas where tradition placed a greater emphasis on the family unit, the home care staff seemed to consciously or unconsciously shape the care so it adapted to the individual’s desire for community. In the urban areas with its high flow of support staff however, more emphasis was placed on just “doing one’s job” and completing certain tasks such as helping the sufferer with evening toilet visits. Less regard was placed on the individual, who found it to be an intrusion into their personal sphere and a threat to their sense of integrity. This could have been resolved with discussions between the caregivers of sufferers and the home care staff providers.

There was a clear pattern showing that caregivers wished to maintain a normal lifestyle in both areas but especially in rural areas. A future challenge could focus on supporting the families and couples in finding a more meaningful and recognizable everyday life for people rather than just covering up the difficulties.

Our findings highlight inherent norms and values constructed in different ways that affect family caregiving, and show how the same caregiver approaches can yield different experiences based on these different constructs. They also correspond to those of Winqvist (1999) on cultural expressions of social class. In this study, informants in urban areas more frequently articulated the need for personal independence while rural informants had a more family-oriented attitude to caregiving.

Our findings show that formal support for caregivers of persons with dementia needs to be more flexible and more tightly targeted to the needs of both the family caregiver and the person with dementia. Support systems often start with the assumption that the optimal solution is to provide full relief for the caregiver. This study demonstrates that such an approach is not consistent with the needs of many caregivers. We have identified the need for greater flexibility and a more nuanced form of support. Family caregivers who demonstrate an acceptance of the course of life and strive to preserve a normal life can be empowered by involving them in decision making, providing professional supervision for active caretaking, and helping them to maintain the family’s sense of unity and closeness. Vikström, Josephsson, Stigsdotter-Neely, and Nygård (2008) found that this eases cooperation between spouses. In some cases, family caregivers are coping well, and it may be sufficient to provide assurance that a range of support services are available should the need arise, reinforcing this during regular visits.

For informants who consider their caregiving as fulfilling an obligation, a range of different support measures are needed in order to reduce the frustrations of providing care. It is important to recognize that there is no single correct answer. Instead, more nuanced support measures need to be formed together with the family caregiver so that these are understandable and recognizable and imbued with an understanding of the families, couple’s and individual’s way of life.

This study demonstrates the importance of a using a more varied knowledge when looking at family life and its influence on approaches to the caregiving experience. By using the social constructive perspective (Berger & Luckmann, 1966/2007) in a caregiving context, we were able to achieve a new understanding of the different sociocultural perspectives that guided the family caregivers’ ways of approaching their situation in rural and urban areas. These results offer insights that can help us to provide more useful and efficient support to these caregivers.

### Limitations

The findings demonstrated transferability by their consistency with other studies in the field of family caregiver experiences. By using an interpretative method, the underlying meanings became visible and enabled us to see differences in approaches that, although relatively subtle, had not been previously identified. It helped us to find variations of family caregiving and supported our assumption that socially constructed cultural traditions of approaching life had an impact on caregiving. The nature of the study and the small sample size limit our ability to generalize the findings, while pointing the way for further research.

To ensure the accuracy and trustworthiness of the interpretation, critical reflection and regular discussions took place between all the authors throughout the entire analysis and writing process. Preliminary interpretations were presented to expert groups at seminars and to the second author.

The hermeneutical dialectical process and the geographical division helped us find contrasts hidden in the language and narratives of the informants. Guided by Gadamer (2002), when analyzing the text, the authors’ pre-understanding was constantly taken into account and discussed in order to be open minded to the text. Quotations were used to allow the reader to participate in the validation and to illustrate the participants’ views.

### Conclusions

This study contributes towards a more nuanced picture of how to view family caregiving by using a hermeneutical dialectic process. Family caregivers who expressed a greater “we” feeling seemed to have a more accepting attitude to the situation and a desire to preserve a normality of life together, while a higher degree of individuality seemed to create more frustration and a greater need for time alone and more freedom of movement. This seems to influence what kind of support is needed. Community-based caregiving needs to develop an awareness of *how* family life and attitudes to caregiving vary and therefore base the support measures accordingly, so that this becomes more in tune with daily life. In line with this, support is needed that provides opportunities for couples and families to maintain a sense of community and togetherness within the home. At the same time, this would also provide relief for caregivers in the form of freedom of movement.
